# Radiomics signature of osteoarthritis: Current status and perspective

**DOI:** 10.1016/j.jot.2023.10.003

**Published:** 2024-03-16

**Authors:** Tianshu Jiang, Sing-Hin Lau, Jiang Zhang, Lok-Chun Chan, Wei Wang, Ping-Keung Chan, Jing Cai, Chunyi Wen

**Affiliations:** a Department of Biomedical Engineering, Faculty of Engineering, The Hong Kong Polytechnic University, Hong Kong SAR, China; b Department of Health Technology and Informatics, Faculty of Health and Social Sciences, The Hong Kong Polytechnic University, Hong Kong SAR, China; c Department of Orthopaedics and Traumatology, Queen Mary Hospital, The University of Hong Kong, Hong Kong SAR, China

**Keywords:** Data mining, Medical image analysis, Osteoarthritis, Radiomics

## Abstract

Osteoarthritis (OA) is one of the fast-growing disability-related diseases worldwide, which has significantly affected the quality of patients' lives and brings about substantial socioeconomic burdens in medical expenditure. There is currently no cure for OA once the bone damage is established. Unfortunately, the existing radiological examination is limited to grading the disease's severity and is insufficient to precisely diagnose OA, detect early OA or predict OA progression. Therefore, there is a pressing need to develop novel approaches in medical image analysis to detect subtle changes for identifying early OA development and rapid progressors. Recently, radiomics has emerged as a unique approach to extracting high-dimensional imaging features that quantitatively characterise visible or hidden information from routine medical images. Radiomics data mining via machine learning has empowered precise diagnoses and prognoses of disease, mainly in oncology. Mounting evidence has shown its great potential in aiding the diagnosis and contributing to the study of musculoskeletal diseases. This paper will summarise the current development of radiomics at the crossroads between engineering and medicine and discuss the application and perspectives of radiomics analysis for OA diagnosis and prognosis.

**The translational potential of this article:**

Radiomics is a novel approach used in oncology, and it may also play an essential role in the diagnosis and prognosis of OA. By transforming medical images from qualitative interpretation to quantitative data, radiomics could be the solution for precise early OA detection, progression tracking, and treatment efficacy prediction. Since the application of radiomics in OA is still in the early stages and primarily focuses on fundamental studies, this review may inspire more explorations and bring more promising diagnoses, prognoses, and management results of OA.

## Graphical abstract

**Figure:**
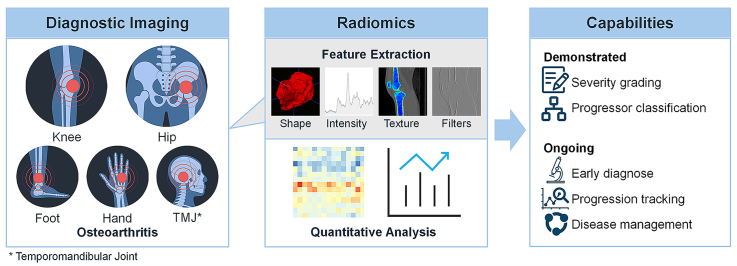

## 1 Unmet need in the precise diagnosis of osteoarthritis

Osteoarthritis (OA) is a prevalent chronic degenerative joint disease mainly affecting the loading-bearing joints such as the knee and hip. It is one of the leading causes of chronic pain and disability in older adults. With the population ageing and obesity pandemic, the prevalence of knee OA has doubled since the mid-20th century [1]. Nowadays, over 500 million people worldwide suffer from OA, causing a heavy socioeconomic burden [2].

Medical images play a crucial role in the diagnosis and prognosis of OA patients. They can be captured through multiple modalities, such as X-ray, computed tomography (CT), and magnetic resonance imaging (MRI). However, each modality has its own strengths and weaknesses in detecting OA [3]. For example, X-ray is a convenient and cost-effective imaging tool for clinically diagnosing radiographic OA, but it is a 2D projection with overlapping signals. It also fails to provide any information on cartilage. A CT scan can accurately display bone structures in 3D, but similar to X-ray, it cannot reflect soft tissues. Besides, the high dose of radiation prevents its application for early screening. MRI is a powerful imaging modality that has superior contrasts on soft tissues. However, routine MRI is less feasible due to its high cost and low accessibility. In addition, artifacts such as distortions in bone structure may limit its accuracy.

Although joint space narrowing, osteophytes, and subchondral bone changes are considered radiographic evidence of OA routinely, these findings are present in the late stage of the disease [4]. Furthermore, research reveals a considerable portion of mismatch between radiographic OA and symptomatic OA; radiographic evidence of OA does not guarantee to be the source of OA's symptoms, and the absence of radiographic abnormality does not rule out the possibility of symptomatic disease [4,5]. Clinical practitioner, therefore, heavily relies on medical history and physical assessments while diagnosing OA [6], and the result could vary greatly depending on individuals' experiences and interpretations.

In the era of precision medicine, radiomics presents a promising solution for precision OA diagnosis by transforming medical images from qualitative interpretation to quantitative data. Radiomics can extract thousands of quantitative features from medical images that are not readily apparent to the naked eye. These features will be analysed by machine learning models to distinguish different types and stages of OA. This approach may reveal hidden patterns and detect subtle changes, which holds promise for detecting early OA, tracking its progression, and assessing treatment effectiveness.

## 2 Radiomics’ origins and evolving applications

### 2.1 Brief history of radiomics

Radiomics is a rapidly growing field featuring quantitative analysis of medical images. The concept was first introduced by Lambin et al., in 2012 when they proposed a new framework for advanced medical image analysis in oncology that involved extracting a large number of quantitative image features [7]. As an “omics” approach, radiomics leverages multi-perspective quantitative analysis based on various image characterisation algorithms, and the high-dimensional information obtained is usually analysed using machine learning techniques [8]. Over the past decade, radiomics has gained widespread popularity in oncology research due to the growing availability of high-quality shared databases, advancements in machine learning and deep learning algorithms, and increased access to high-performance computational hardware. Furthermore, it has demonstrated promising potential as a tool for precision medicine, with the ability to support accurate diagnosis, prognosis, and predictions of therapeutic outcomes [9].

### 2.2 Radiomics analysis pipeline

**Figure 1:**
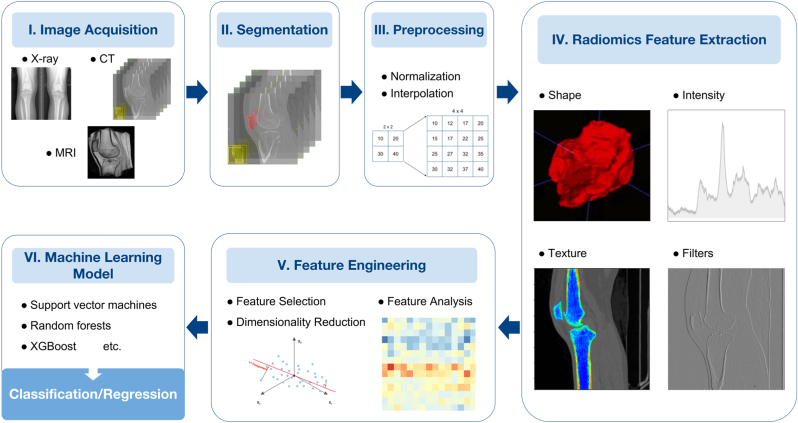
Overview of radiomics analysis framework.

A typical radiomics analysis pipeline includes several steps, including image acquisition, image pre-processing, segmentation, feature extraction, dimension reduction, and model development, as shown in Fig. 1. Radiomics analysis can be performed on medical images acquired using various modalities, such as X-ray, CT, and MRI. Due to the heterogeneous image acquisition parameters in a clinical setting, image pre-processing procedures such as resolution harmonisation and intensity normalisation are preferred to improve reproducibility and stability. Segmentation defines the region of interest (ROI) in 2D images or volume of interest (VOI) in 3D images for feature extraction. It can be either conducted manually or by automatic segmentation tools. Pre-defined radiomics features are then calculated within the VOI/ROI from either original or filtered images (e.g., Laplacian of Gaussian, wavelet filters).

Various classes of radiomics features have been proposed and standardised by the Imaging Biomarker Standardization Initiative (IBSI) in 2016 [10]. They include shape-based features describing the size and shape of the VOI/ROI, first-order features describing histogram-based statistics of voxel intensity, gray level co-occurrence matrix describing the probability of similar pixels joining together, gray level run length matrix describing the length of consecutive pixels of the same grey level, gray level size zone matrix describing the number of connected voxels with the same grey level intensity that forms grey level zones, gray-tone difference matrix describing the texture from fractal properties to quantify the difference between a gray value and the average among neighbouring voxels, and gray level dependence matrix measuring grey level dependency on the centre voxel of voxels in a region. The number of features extracted can exceed one thousand, leading to a much higher dimensionality than the sample size. As a result, feature selection or dimension reduction techniques using machine learning or statistical methods are necessary to reduce redundancy and avoid false discoveries. The selected independent and informative radiomics features are combined with a mathematical model for the final classification or regression task.

Moreover, a well-designed radiomics analysis shall take multiple factors into consideration to ensure the successful application of radiomics. Variability in image acquisition parameters, such as imaging protocols and scanners, can introduce inconsistencies in the extracted radiomic features for the same disease condition. The reproducibility of feature extraction is also influenced by algorithm robustness, manual or automatic segmentation accuracy, and parameter choices related to preprocessing settings, algorithmic thresholds, and segmentation criteria. Additionally, data quality issues like image artifacts and noise, along with institutional variabilities, can affect the reliability of radiomic features. The establishment of comprehensive guidelines with standardized protocols for each procedure of the radiomics pipeline shall facilitate result comparison and foster the clinical applicability of radiomics analysis [11].

While a typical radiomics analysis follows the analysis pipeline mentioned above, this review will use a broader definition and consider quantitative analysis involving any mathematically defined features extracted from medical imaging as radiomics.

### 2.3 Evolving applications of radiomics

Radiomics’ high-throughput extraction of image features enables data mining from a significant amount of information regarding the disease presentation in medical images, a large portion of which could be unique from the results of clinical assessments and laboratory tests [12]. Such independent information may improve prognosis and therapeutic response predictions, leading to personalised treatment plans and more efficient clinical workflows. As a result, radiomics is utilised to build automated pipelines to assist clinical decision-making on various diseases. Radiomics has been extensively investigated in the field of oncology [13], encompassing the diagnosis of cancers in diverse body tissues, prognostication of recurrence risk, patient survival, metastatic potential, treatment response, the prediction of molecular characteristics among cancer patients, and even treatment planning [14,15]. Excitingly, diseases beyond cancer have begun to witness active exploration, such as coronavirus disease 2019 [16], cardiovascular disease [17], liver fibrosis [18], and OA.

Presently, OA in medical images is analysed using clinically pre-defined features like the Kellgren and Lawrence Grade (KL Grade), MRI Osteoarthritis Knee Score (MOAKS), or Whole-organ magnetic resonance imaging score (WORMS). Based on radiographic measurements or observations, these scales offer direct and intuitive disease interpretation but are often criticised for their low precision and high inter-observer variability. Deep learning-based approaches have also been explored, often exhibiting improved performance. However, their lack of interpretability and high learning curves compared to traditional methods pose great challenges for clinical utility. Radiomics could be a potential candidate to achieve a balance by combining the computational power of machine learning with the interpretability of hand-crafted features [19], thus bringing increasing applications in OA diagnosis and prognosis [20].

## 3 Current applications of radiomics in OA

### 3.1 Applications of radiomics for OA research

Before a systematic radiomics approach was applied in OA research, many studies performed image data mining by extracting multiple mathematical features. They are still recognised as radiomics studies and included in this review, as similar quantitative analyses were performed in OA detection, classification, and prediction. In the recent three years, several studies achieved enhanced predictive performance by leveraging multidimensional radiomics features followed by standard radiomics analysis steps. With the increasing popularity of radiomics in the OA research community, various joints that are commonly affected by OA, such as the hand/wrist, hip, knee, ankle/foot, and temporomandibular joint (TMJ), have been studied (Table 1).

**Table 1:** Radiomics applications of various OA joints based on different imaging modalities.

Application	X-ray	CT Scan	MRI
Classification	Knee OA^a^ – Healthy vs OA [[21], [22], [23], [24], [25]] Knee & Hand OA – Healthy vs OA [26]	TMJ^b^ OA – Healthy vs OA [27,28] Hand OA – Young vs Older [29]	SpA^c^ – Healthy vs OA [30] Knee OA – Healthy vs ACLR^d^ [31] Knee OA – Healthy vs OA [[32], [33], [34], [35], [36]] Ankle OA – Dancer vs Normal Person [37]
Detection	Knee OA – Disease Grade [38] Hand OA – Osteophyte [39]	Ankle OA – Deformity Characteristics [40]	Knee OA – Disease Grade [41]
Prediction	Knee OA –Progression vs Non-progression [21,42] Knee & Hand OA – Progression vs Non-progression [26] Hip OA – Risk of Incident [43]	TMJ OA – Risk of Incident [44]	Knee OA – Treatment Response of Vitamin D [45] Knee OA – Pain Improvement [46] Knee OA – High Risk vs Low Risk [41] Knee OA – Risk of Incident [47,48]

a OA involving osteoarthritis

b TMJ involving temporomandibular joint

c SpA involving spondyloarthritis

d ACLR involving anterior cruciate ligament reconstruction

Compared to traditional clinical variables, radiomics has demonstrated its superior capabilities in detecting patellofemoral OA of the knee joint [22] in one study that analysed 5507 knees with 17.3 % PFOA prevalence. This study utilised a landmark detection tool, BoneFinder, to automatically identify and analyse the patellar ROI using hand-crafted features and convolutional neural networks (CNN). The findings underscore the superior performance of texture features, which achieved a higher area under the receiver operating characteristic curve (AUC) than conventional clinical variables. This breakthrough in the classification of PFOA highlights the potential of radiomics and texture analysis in refining our diagnostic approach, possibly leading to earlier and more precise disease detection.

The standard radiomics analysis has shown considerable advantages among various texture analysis methods. This is evidenced by several studies conducted on TMJ OA by a U.S. research group using diverse methodologies [28,44,49,50]. Their latest work [44] attained the highest performance through radiomics. They extracted and selected 13 radiomics features from CT of 92 patients that showed statistically significant differences between the OA and control groups on the TMJ. The radiomics features were combined with clinical biomolecular variables into a total set of 52 biomarkers and modelled by different machine learning techniques. After comparing models developed by Logistic Regression, Random Forest, LightGBM, and XGBoost, the best machine learning model, “XGBoost + LightGBM”, achieved 87.0 % of the AUC for diagnosing TMJ OA status.

Combining radiomics features with other features has been demonstrated to further improve performance. For example, one research group extracted principal components analysis (PCA)-based shape, radiomics-based texture, and appearance features from lateral knee radiographic images [21]. They applied indecisive trees to distinguish between healthy and OA people's radiographs and predict whether people who are healthy at baseline would develop OA within 84 months. The study used the MOST dataset with a large sample size and combined shape and texture features to achieve 0.88 AUC in the classification. Similarly, shape features derived from Statistical Shape Model and radiomics features were combined from knee joint X-rays to detect OA automatically [38]. The best performance was achieved when combining shape and texture features compared to individual feature categories.

Despite the promising performance of radiomics in the detection, prediction, and classification of OA demonstrated by existing literature (Table 1), limitations still exist that prevent further clinical applications in OA.

OA is a complex and prolonged disease, but most current studies have only been able to achieve binary diagnosis results, and limited studies have investigated more detailed disease presentations using quantitative approaches. For instance, a significant association was found by comparing bone trabecular texture features in X-rays of finger joints with MRI-defined bone flab information within 21 female OA patients [39]. Such a relationship suggests that the textural of bone trabeculae may contribute to the early detection of OA in hand. Although this study provides a possible direction for the research in early disease detection, the subject amount is too small to validate the findings. Another notable study has effectively quantified and predicted the effect of vitamin D in the treatment of OA using MRI-based radiomics features [45]. A total of 216 patients were enrolled in the study, and radiomics features were extracted from baseline MRI scans to predict the treatment response. The researchers further categorised the efficacy of the OA treatment response to vitamin D into two groups based on a 20 % improvement in knee pain over a 48-h period. This research is one of the few current studies that have a relatively solid conclusion regarding prognosis, employing a standard radiomics process with sufficient subjects.

There are other limitations to the current study on OA. The affix “osteo” in osteoarthritis highlights the importance of subchondral bone in the pathogenesis and management of OA. CT scans provide a relatively clear shape, texture and structure of bones, but currently, few studies have analysed the CT presentation of subchondral bone for OA diagnosis, especially knee OA. Moreover, more comprehensive radiomic analysis procedures may increase the sensitivity of OA predictions compared to considering only one or two image characteristics, such as gray level co-occurrence matrix and first order features. Cross-institutional validation is also required to guarantee stability and generalizability, especially for studies with small sample sizes. However, many studies are missing external validations due to challenges such as regulations and data security concerns. The lack of transparency and standardisation in data processing and feature extraction also prevents third-party validations.

### 3.2 Applications of radiomics for OA-related musculoskeletal tissue research

OA is a complex condition that affects multiple components of the joints. The involvement of various tissues in the joint contributes to the complexity of the disease diagnosis and treatment. Fortunately, a growing number of studies have been focusing on the applications of radiomics-based medical image analysis on various OA-related tissues, including that of cartilage [51,52], ligaments [53], tendon [54,55], bone [[56], [57], [58]], muscle [59,60], fat, etc., as summarised in Table 2.

**Table 2:** Radiomics applications of various OA-related tissues.

Tissue	Year	Target	Imaging Modality	Patients (Number)
Cartilage	2022	Detect the degree of meniscus injury [51]	MRI^a^	152
	2021	Classify atypical cartilaginous tumours & appendicular chondrosarcomas [52]	CT^b^	120
Ligament	2021	Predict Anterior Cruciate Ligament rupture [53]	MRI	68
Tendons	2022	Diagnose Achilles Tendinopathy [54]	Ultrasound	139
	2016	Recognise rotator cuff supraspinatus tendon tear [55]	Ultrasound	40
Bone	2022	Detect osteoporosis of the lumbar spine [56]	CT	133
	2020	Classify osteoporosis, osteopenia, and normal patients [57]	DXA^c^	147
	2019	Predict metastasis risk of osteosarcoma [58]	PET^d^/CT	83
Muscle	2021	Identify sarcopenia [59]	CT	247
	2021	Classify idiopathic inflammatory myopathies [60]	MRI	74
Fat	2021	Predict type 2 diabetes mellitus & metabolic syndrome status [61]	MRI	310
	2021	Evaluate metabolic disorders & Predict surgery-induced weight loss effects [62]	CT	675

a MRI involving magnetic resonance imaging

b CT involving computerised tomography

c DXA involving dual-energy X-ray absorptiometry

d PET involving positron emission tomography

The imaging properties of each musculoskeletal tissue vary based mainly on composing materials and physical characteristics; therefore, different imaging modalities could be adopted for capturing interested pathological information. The advantage of radiomics analysis is the ability to exhaustively extract and analyse a vast number of quantitative features regardless of the type of tissue, disease type or image modality [12], even those specialised in functional information such as dual-energy X-ray absorptiometry (DXA) [57], positron emission tomography (PET)/CT [58], etc.

Some of the most recent findings of cartilage radiomics focused on sarcomas from CT images [52] and tissue degeneration from dual-mode MR images, providing a non-invasive alternative to traditional diagnostic practices with comparable performance. For tendons, ultrasonography radiomics were investigated for detecting tissue injury [54,55], which outperformed manual readings from physicians in clinical settings thanks to its quantitative and objective nature. Muscle radiomics were adopted to identify degenerative diseases [59] and autoimmune disorders [60] from CT and MRI, respectively, showing how radiomics are applicable to distinct pathological processes even among the same type of body tissue. Radiomic features of fat tissues extracted from MR [61] and CT [62] images were also demonstrated to predict metabolic disorders. Given that metabolic alterations are often biomarkers for early disease diagnosis, these findings enlighten the future developments of imaging biomarkers for early disease diagnosis and prognosis through effective data mining. At the same time, these findings also provide indirect evidence of the prospective use of radiomics in the study of osteoarthritis.

## 4 Perspectives on the future of radiomics in OA

### 4.1 Prospects of radiomics in OA

The rising prevalence of OA among ageing populations highlights an urgent need to develop accurate and efficient diagnostic tools. This necessitates exploring innovative methodologies, such as radiomics, to enhance detection, classification, and prediction capabilities for OA. Several studies have demonstrated promising results by utilising only one or two classes of radiomics features. Other studies have shown that radiomics features perform well in combination with other features (clinical, demographics, etc.) for precise diagnosis and prognosis of OA. Furthermore, a deep learning-based radiomics approach can directly generate and identify quantitative image features from input images in the neural network's hidden layers without image segmentation and pre-defined mathematical formulas for feature calculation [12,14]. The framework holds great promise for future radiomics research in the diagnosis and prognosis of OA.

Moreover, radiomics may facilitate early diagnosis by reflecting subtle changes that conventional diagnostic methods may not identify. While limited studies have demonstrated radiomics in early diagnosis, further research is needed to explore its accuracy in various image modalities and fulfil diverse clinical requirements. For instance, while MRI is more accurate than X-ray in early OA detection [63], its high cost and time consumption often lead to the preference for using radiography. It remains to be seen whether the combination of radiomics and radiography can achieve comparable accuracy of early detection to MRI. Meanwhile, early diagnosis of OA may lead to overdiagnosis and overtreatment, especially for patients whose disease progresses slowly or not at all [63]. Therefore, it is crucial to investigate the disease progression prediction tasks to establish personalised disease management [64], such as estimating disease trajectory, evaluating the risk of complications, assessing response to treatment, and modelling survival outcomes.

With the standardised feature definitions by IBSI, radiomics may be a solution to the major challenges in terms of cross-institution validation. Other obstacles, such as inconsistent datasets, diverse study designs, and varied data processing and analysing procedures, have significantly hindered advancements in image-based OA diagnosis and prognosis predictions. Recognizing the need for reliable datasets for clinical development, the community has initiated large-scale OA image databases like the Osteoarthritis Initiative (OAI), Multicenter Osteoarthritis Study (MOST), and Cohort Hip and Cohort Knee (CHECK). These databases, each containing thousands of samples, pave the way for the development of more reliable models and enable large-scale cross-institutional validations, bringing radiomics closer to daily clinical application. Nevertheless, clinical applications of radiomics are still rare [65], possibly due to the low efficiency and poor transparency in data processing and model establishment. Prospective studies can provide stronger evidence of cause-and-effect relationships, which reduce the possibility of false discovery and enhance the reliability of the findings. Such research is needed in the future to verify the clinical capabilities of radiomics.

### 4.2 Recommendations for applications of radiomics in OA

While radiomics has shown potential for OA diagnosis and prognosis, there are several limitations and challenges to its widespread clinical application. The following considerations should be taken into account to ensure successful radiomics applications for OA:

#### 4.2.1 Imaging modality

Each imaging modality serves a unique purpose in a patient's diagnosis and treatment journey. Different imaging modalities may be utilised to obtain specific information at different stages of disease management. For instance, X-rays are used for screening, while CT provides detailed bone structure information before surgery. Currently, most radiomics research has focused on MRI, while other imaging methods, such as CT and radiography, may contain unique information and deserve further investigation. The harmonisation of imaging protocols for each of the imaging modalities is crucial to improve the comparability and generalizability of radiomics studies. This includes specifying acquisition parameters, image reconstruction techniques, and quality control measures.

#### 4.2.2 Sample size

Medical images can be difficult to access due to large data volume and patient privacy concerns, resulting in smaller sample sizes for retrospective studies. However, a small sample size may increase the risk of overfitting the constructed models, resulting in biased and unreliable prediction outcomes. To address this issue and ensure the robustness of the model, it is recommended that a sample size of at least 100 data per group be used after appropriate quality screening in radiomics analysis [8].

#### 4.2.3 Feature selection

During feature extraction, a radiomics study may generate thousands of features. This high-dimensional feature space of sparse data increases the risk of false discovery and computation cost, making feature selection a critical step in radiomics analysis. As a rule of thumb, the number of retained features should be no more than one-tenth of the sample size [66]. Additionally, it is important to consider the reproducibility and generalizability of selected features in light of varying image quality, resolution, and scale from different operators, equipment, and institutions. Test-retest and phantom studies can be utilised to address these issues [67,68].

#### 4.2.4 ROI/VOI selection

The region selected for feature extraction (ROI/VOI) can greatly impact prediction sensitivity. For example, features extracted from the medial subchondral bone region of the knee have shown a higher association with OA than those from the lateral subchondral bone [32]. Due to the varying information that radiomics features contain in different locations, it may be beneficial to compare several different ROIs/VOIs and select the best region for analysis [24,32].

## 5 Conclusion

The increasing prevalence of OA calls for the development of more accurate and efficient diagnostic tools, especially for early detection, intervention, and treatment. Radiomics offers high accuracy and interpretability in the analysis of medical images, making it a valuable tool in the diagnosis and prognosis of OA. While limited studies have demonstrated the accuracy of radiomics in predicting OA progression, further research is needed to evaluate its early detection ability and fulfil clinical requirements. Moreover, the standardised feature definitions and large-scale OA image databases enable reliable modelling and cross-institutional validations, leading the way towards daily clinical application. Future radiomics research in both the diagnosis and prognosis of OA are warranted to further address medical and social needs of OA patients.

## Funding

This study is supported by SZRI start-up (I2021A013) and RISports Seed Fund (P0043526), Projects of RISA (P0043001, P0043002), Project of 10.13039/501100022097RIAM (P0041372), and Project of Strategic Importance Fund (P0035421) of the 10.13039/501100004377Hong Kong Polytechnic University.

## Authorship

Conception and design of study: Tianshu Jiang, Ping-Keung Chan, Jing Cai, Chunyi Wen, acquisition of data: Tianshu Jiang, Sing-Hin Lau, Lok-Chun Chan, Ping-Keung Chan, analysis and/or interpretation of data: Tianshu Jiang, Sing-Hin Lau, Jiang Zhang , Jing Cai, Drafting the manuscript: Tianshu Jiang, Sing-Hin Lau, Jiang Zhang, revising the manuscript critically for important intellectual content: Lok-Chun Chan, Wei Wang, Jing Cai, Chunyi Wen, Approval of the version of the manuscript to be published: Tianshu Jiang, Sing-Hin Lau, Jiang Zhang, Lok-Chun Chan, Wei Wang, Ping Keung Chan, Jing Cai, Chunyi Wen.

## Declaration of competing interest

A conflict of interest occurs when an individual's objectivity is potentially compromised by a desire for financial gain, prominence, professional advancement or a successful outcome. The Editors of the *Journal of Orthopaedic Translation* strive to ensure that what is published in the Journal is as balanced, objective and evidence-based as possible. Since it can be difficult to distinguish between an actual conflict of interest and a perceived conflict of interest, the Journal requires authors to disclose all and any potential conflicts of interest.
